# Overlooked: Extrachromosomal DNA and Their Possible Impact on Whole Genome Sequencing

**DOI:** 10.21315/mjms2018.25.2.3

**Published:** 2018-04-27

**Authors:** Reinhard H Dennin

**Affiliations:** Department of Infectious Diseases and Microbiology, University of Luebeck, UKSH, Campus Luebeck, D-23538 Luebeck, Germany

**Keywords:** extrachromosomal, episomal, genome, sequencing, chromosome

## Abstract

Extrachromosomal (ec) DNA in eukaryotic cells has been known for decades. The structures described range from linear double stranded (ds) DNA to circular dsDNA, distinct from mitochondrial (mt) DNA. The sizes of circular forms are described from some hundred base pairs (bp) up to more than 150 kbp. The number of molecules per cell ranges from several hundred to a thousand. Semi-quantitative determinations of circular dsDNA show proportions as high as several percentages of the total DNA per cell. These ecDNA fractions harbor sequences that are known to be present in chromosomal DNA (chrDNA) too. Sequencing projects on, for example the human genome, have to take into account the ecDNA sequences which are simultaneously ascertained; corrections cannot be performed retrospectively. Concerning the results of sequencings derived from extracted whole DNA: if the ecDNA fractions contained therein are not taken into account, erroneous conclusions at the chromosomal level may result.

## Introduction

Extrachromosomal dsDNA (ecDNA)–apart from the mtDNA–in eukaryotic cells of human, animal, and plant origin has been known for more than three decades at least (1–5). These ecDNA molecules are, according to the structure, both linear and circular dsDNA; the circular ones are mostly termed small polydispersed circular DNA (spcDNA); occasionally synonymously termed extrachromosomal circular DNA (eccDNA) just to classify the DNA structure (6, 7). They differ in size from about several hundred base pairs (bp) (8) up to ≥ 25 kbp, and up to more than 150 kbp (9) for the large circular sized DNA. The number of spc/eccDNA molecules ranges from several hundreds up to one thousand per cell. The term “polydispersed” already hints at a great diversity of these DNA molecules; it may also indicate different types of functions. The spc/ecc-DNA fraction can constitute a substantial part of the whole DNA extracted, for example from human peripheral blood mononuclear cells (PBMCs). Semi-quantitative experimental assessments show the spc-dsDNA fraction constitutes several percentages of the whole DNA extracted from PBMCs of healthy human subjects (10). However, it may depend on, for example, the metabolic status of the cell’s activation. Regarding certain sequences, there are indications for “rolling circle replication” (11). Therefore, the composition of ecDNA might change with time and the sequences could be different upon replication. Circular DNA forms derived from exogenous viral infections such as Hepatitis B virus (HBV) (12) or retroviral E-DNA (13) are not considered here.

There is broad agreement that ecDNA originates through diverse mechanisms, for example mobile genetic elements (MGE) (14) and “Mismatch Repair Pathways” (15), that can rearrange diverse DNA sequences from chrDNA (16). In the context of mobile DNA, different kinds of retro-/transposons (17, 18), long interspersed nucleotide elements (LINE) (19), short interspersed repetitive DNA sequences such as Alu elements (20), and telomeric repeats (21) are known to be essential parts of chrDNA. They are also detected in the ecDNA fraction of eukaryotic cells. This means that chromosomal derived sequences are present in the ecDNA but rearranged in some way; how this works in detail remains speculative (22). Checking these mobile activities revealed aspects of both randomly and non-randomly caused instabilities within the genome (23). It may be based on intrinsic, genetically ingrained structures that are activated on demand by environmental impacts. This might reflect the plasticity of the human genome (24), including the ecDNA. Apart from general aspects, functions of ecDNA in different eukaryotic cells are not known. Yet, certain hints concerning cancer cells are given as described in section ‘4’ below.

It is not known for sure: (i) where the formation of ecDNA takes place, in the cell nucleus and/or the cytoplasm, and (ii) whether it is a short transitory formation or a long time stable status of certain ecDNA regarding possible metabolic functions. Protocols for the preparation of ecDNA, in particular ecc-dsDNA, are outlined by a few studies (22, 25, 26). The reported findings of sequences homologous to chrDNA in ecDNA may reflect the particular interests of the authors and what they were looking for; their results may represent only a tiny part of the real existing sequences of chromosomal origin contained in ecDNA with yet unknown impact. It seems realistic to assume that the extensive repertoire of ecDNA sequences contains even more different chromosomal sequences than previously known.

### Possible Impacts

Four selected situations should draw attention to possible discrepancies when “whole DNA from eukaryotic cells” was used for genome sequencing (whole genome sequencing, WGS) to ascertain the sequences of the respective chromosomes. They are intended to point to a paramount importance of both general as well as specific aspects of genetics.

1. General aspects: The formation of ecDNA is obviously a complex process. However, it is known that ecDNA are composed by means of chromosomal mobile sequences, such as transposons, long terminal repeats (LTR), and Alu elements, but they themselves contain these sequences in different degrees. Therefore, in addition to the existence of ecDNA itself, a potential for changes in the composition of their sequence, including an active function, cannot be ruled out. This concerns species of ecDNA, for example those showing sequences in coincidence with Human Endogenous Retro-Viruses H (HERV-H) (27). HERVs have the potential to replicate and to transpose themselves. Therefore, when discussing the issues of HERVs based only on results from sequenced human genomes based on whole cellular DNA, the possible “contaminating influence” of ecDNA with integrated HERV sequences remains unmentioned (28). Studies on, for example endogenous viruses (29), LINE, *et cetera*, performed with whole DNA extracts from human cells or tissues may need to be reevaluated because the ecDNA fractions were not taken into account. This uncertainty is because sections of the LTR sequences of HERVs have been depicted to be present in the spc/ecDNA fraction. Studies on retrotransposons (30, 31) should be reconsidered regarding their allocation upon alignments of their ecDNA and/or chrDNA if sequencing data is based on whole DNA extracts only. These considerations apply to plants too (32). Therefore, vagueness remains as these facts have not yet been considered in otherwise exceptionally structured review articles (33).
2. The human genome project: Basically, the same holds true for sequencing projects regarding the human genome (34). Here, the term “genome” usually pertains to chrDNA in eukaryotic cells. When the extracted whole DNA from the respective human cells was used for fragmentation, cloning, and sequencing (35, 36), it means: the whole DNA was not separated into (i) chrDNA and (ii) ecDNA before fragmentation. That is, the fragments generated for sequencing consist of a mixture of short DNA sequence stretches derived from both chrDNA and ecDNA, apart from the well-known mtDNA. Furthermore, if the fragmentation of the whole DNA was performed with restriction endonucleases, the remaining single stranded ends of their cutting sequences from fragments derived both from chrDNA and ecDNA would be identical. An incorrect assignment of fragments originating from the ecDNA into the final chrDNA is, therefore, more likely. In addition, the possible different patterns of methylation of the cutting sequences of the applied restriction enzymes on both chrDNA and ecDNA sequences might result in uncertainties on alignments of chrDNA. Similarly, findings indicate that using mechanical shear forces on whole DNA to get fragments for next-generation sequencing (NGS) results in DNA sequences that are non-randomly fragmented (37). This effect has not been considered for ecDNA with respect to their broad range in molecular sizes. The various ecDNA contain rearranged DNA or DNA composed of shorter chromosomal sequences, for example by incremental acquisition by MGEs. Depending on the proportion of ecDNA in the subject’s whole cell DNA, a greater or lesser extent of sequences of ecDNA was fragmented, cloned, and subjected to NGS. Sequences derived from ecDNA do not carry tags that exactly predict their source, such as derived from ecDNA, and might have been handled as sequences of chromosomes in the final alignments. NGS of the whole DNA of eukaryotic cells allows no discrimination between chrDNA and ecDNA sequences. Nothing is known about how many, and where, ecDNA derived sequences are wrongly placed into chrDNA; this may have led to wrong conclusions in final analysis in various fields. Therefore, critical aspects arise: possible uncertainties with the allocation and alignment of the final chromosomal sequences have to be taken into account if the ecDNA fractions have not been considered.
3. The ENCODE project: Furthermore, the issues addressed also apply to discussions on epigenetics of the human genome (38). The ENCODE project is designed to look for epigenomes in the human genome, for example (39). However, ecDNA could exhibit patterns of individual methylation too (10). Therefore, they might be co-precipitated, for example, according to the immunoprecipitation protocols for methylated DNA. These possible situations have not been considered in the respective results.
4. Medical aspects: “…as next-generation sequencing begins to break down the barriers between research and the clinic, as genomic and clinical data are responsibly integrated into the “cloud” to look for patterns of health and disease…” (33). However, it is imperative to note that results showing increased numbers of ec/spcDNA in aged and malignant cells containing indicator sequences (40), in particular the double-minute ecDNAs (41, 42), must receive attention. In cancer cell lines, the amount of spcDNA can be as high as up to 17.8% (14). The points in question here are the issues: when sequencing whole DNA from such aged cells, how do these indicator sequences harbored in ecDNA get identified for the final chromosomal sequence alignments? How are they treated if they contain mutations, single ones or multiple copy number variations? Not considering the fraction of ecDNA in the case of the individualised sequencing of whole DNA for multigenic analysis may entail incorrect association in disease assessments. This has also to be seen in the context of Alu sequences in “germline genetic diseases” (43), which also applies to “normal lymphocytes” (14). Therefore, activities to integrate patients’ genetic data gained from NGS of whole DNA into this “cloud” to use them as diagnostic tools should not be enforced until any uncertainties possibly deriving from ecDNAs are eliminated.

## Discussion

Studies on ecDNA from human cells and tissues have shown that they contain genetic elements which are known to belong to chrDNA. Protocols are available for the correction of possible “sequence errors” after sequencing (44–48). However, genomic DNA sequencing, that is WGS, in which ecDNA has not been separated from chrDNA prior to fragmentation can cause problems. This applies if sequencings are performed and the results are analysed without knowledge of the sequences of the ecDNA as parts of the whole cellular DNA. It is, therefore, questionable whether, after NGS of the whole DNA of eukaryotic cells, corrections to chrDNA with bioinformatics lacking data on ecDNA can be adequately achieved.

Therefore, evidence-based studies are required to demonstrate whether the disregard of ecDNA can or may not cause any uncertainties in the assessment of chromosomal sequences in general, and also of diseases on the basis of individualised sequencing. For example, sequences of ecDNA have to be subtracted by correctional algorithms from sequences concurrently obtained from whole DNA of cells to keep chromosomal sequences free from contaminating ecDNA sequences. Further strategies for that reason are outlined by a few studies (22, 25, 26).

The demand for evaluating NGS results using “professional standards” is correct (49), but it requires additional aspects due to possible imprecisions by ecDNA. This also applies to the latest NGS (50, 51), especially when cancer neochromosomes have to be considered (52). Based on such results, it should be possible (i) to clarify the potential impact of hitherto not considered ecDNAs on whole DNA sequencing, (ii) to exclude imponderabilities as far as possible, and (iii) to develop standards that make the results of different NGS protocols undoubtedly comparable.

## Conclusions

This review raises critical questions about the reliance on so-called genome sequencing data of whole DNA, in particular of human genomes. EcDNA in eukaryotic cells is a fact. The evaluation of their possible impact in NGS of the human genome is necessary. Independent of the sequencing protocols applied, if the ecDNA has not been separated from chrDNA prior to fragmentation, uncertainties may remain regarding the final chromosomal sequences depending on the fraction of admixtures from ecDNA. Certain aspects have to be addressed: (i) the self-replicating potential of ecDNA with the consequence of mutations and rearrangements that can result, (ii) possible interchange in both directions between ecDNA and chrDNA. Careful assessments of the risks are necessary, especially when it comes to medical applications.

## Abbreviations

Alu: sequences named according to the restriction endonuclease AluI
bp(s): base pair(s)
chrDNA: chromosomal DNA
ecDNA: extrachromosomal DNA
eccDNA: extrachromosomal circular DNA
HERV-H: Human Endogenous Retroviruses type H
mtDNA: mitochondrial DNA
LINE: long interspersed nucleotide elements
LTR: long terminal repeats
MGE: mobile genetic element(s)
NGS: next-generation sequencing
spcDNA: small polydispersed circular DNA
PBMC: peripheral blood mononuclear cells
WGS: whole genome sequencing.
